# Supplementary figures and images for: Mono-mix strategy enables comparative proteomics of a cross-kingdom microbial symbiosis
Source: PLoS One. 2026 Jan 16;21(1):e0340253. doi: 10.1371/journal.pone.0340253 (PMC12810790; doi:10.1371/journal.pone.0340253)

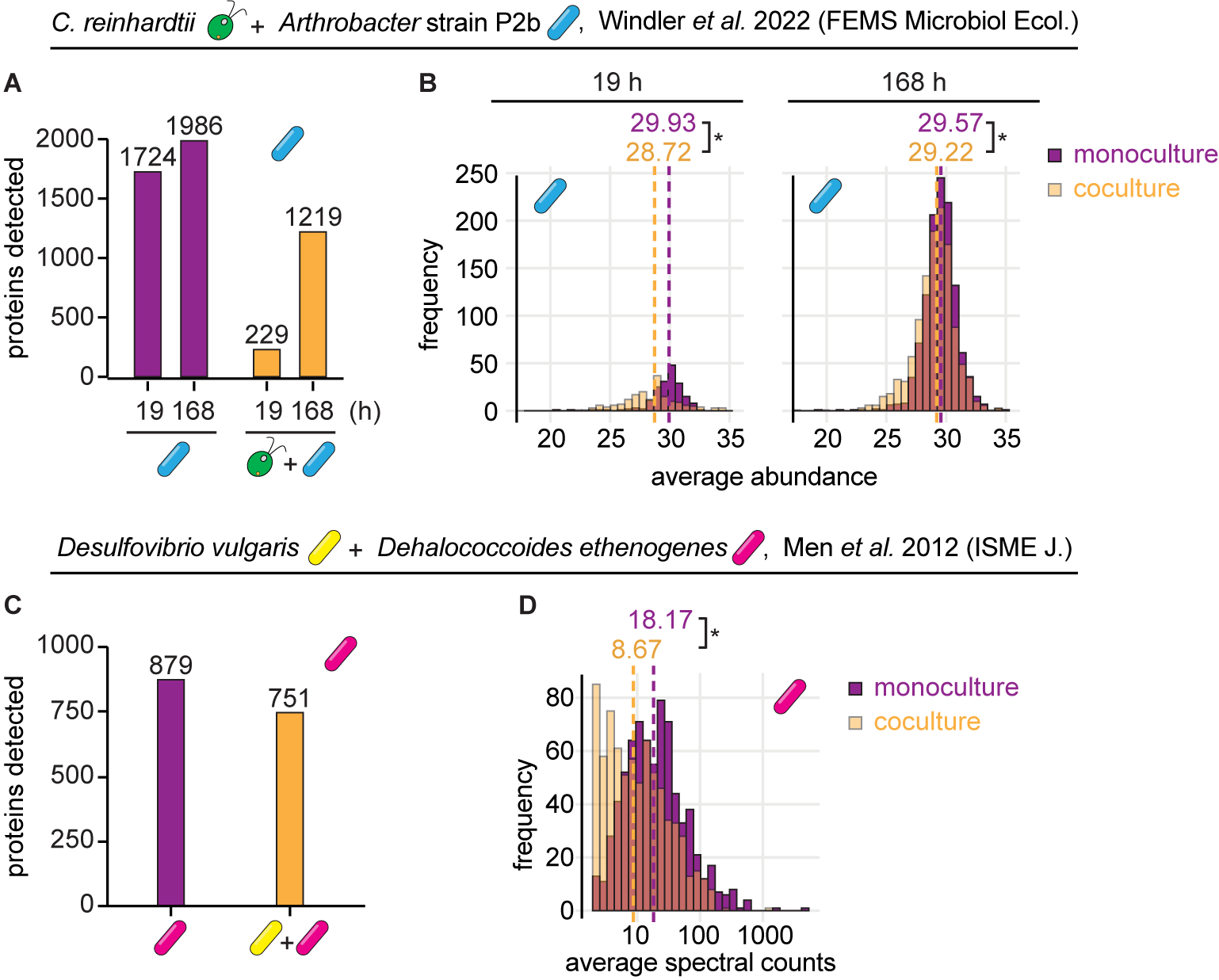

Supplement: S1 Fig — (A) Number of unique Arthrobacter proteins with at least two peptides detected in at least one biological replicate of monocultures (purple) and cocultures with C. reinhardtii (orange) 19 h and 168 h after inoculation by LC-MS/MS proteomics reported in Windler et al. 2022 [21]. Spectra were mapped to the C. reinhardtii v5.6 genome annotation (17,741 protein-coding genes) and Arthrobacter strain P2b genome annotation (4,475 protein-coding genes). (B) Distribution of Arthrobacter protein abundances in monocultures (purple) and cocultures with C. reinhardtii (transparent orange) for proteins detected in both conditions reported in Windler et al. 2022 [21]. When distributions overlap, the color is muddied. Median values are shown above the dashed lines. Asterisks next to the medians indicate significant differences between the indicated distributions by a Wilcoxon rank-sum test (p < 0.05). (C) Number of unique Dehalococcoides ethenogenes proteins detected in at least two biological replicates of monocultures (purple) and cocultures with Desulfovibrio vulgaris (orange) by LC-MS/MS proteomics reported in Men et al. 2012 [54]. Spectra were mapped to a concatenated database of amino acid sequences from D. ethenogenes strain 195 (~1,500 protein-coding genes) and D. vulgaris strain Hildenborough (~3200 protein-coding genes). (D) Distribution of D. ethenogenes protein abundances in monocultures (purple) and cocultures with D. vulgaris (transparent orange) for proteins detected in both conditions reported in Men et al. 2012 [54], presented as in (B). (TIF) [file pone.0340253.s001.tif]

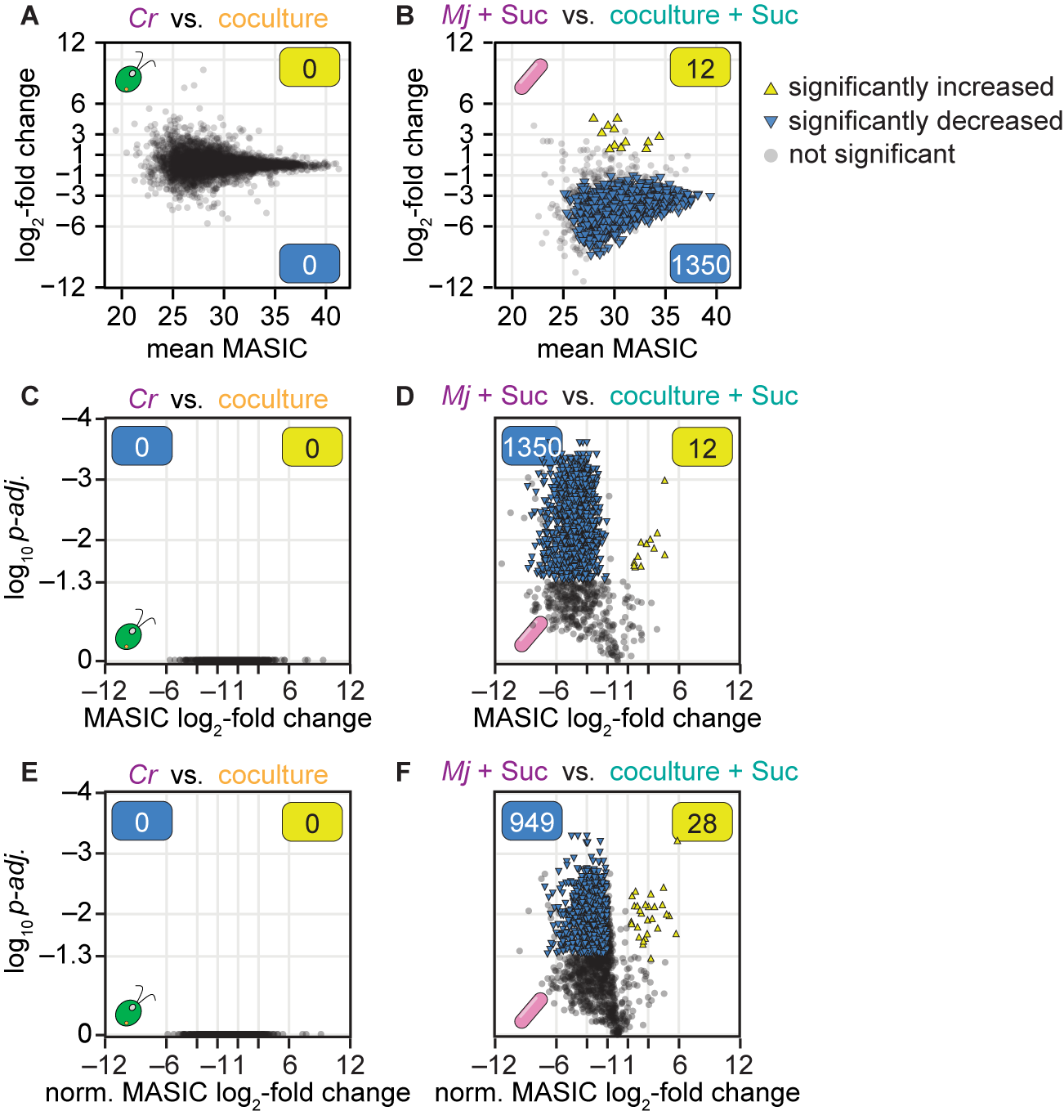

Supplement: S2 Fig — Triplicate continuous-light-grown monocultures and cocultures with and without sucrose were collected for LC-MS/MS proteomics. (A) Changes in unnormalized algal protein abundances in coculture relative to monoculture. Significant differences (triangles) were defined as those where |log2-fold change| > 1, p-adj. < 0.05 from a Welch two sample t-test of the unnormalized MASIC values, and the mean MASIC value was greater than the limit of quantitation in both the coculture and the monoculture. (B) Changes in unnormalized bacterial protein abundances presented as in (A). (C) Changes in unnormalized algal protein abundance in coculture relative to monoculture shown as a volcano plot. Significant differences (triangles) were defined as those where |log2-fold change| > 1, p-adj. < 0.05 from a Welch two sample t-test of the unnormalized MASIC values, and the mean MASIC value was greater than the limit of quantitation in both the coculture and the monoculture. Yellow and blue boxes indicate the number of significantly increased and decreased proteins, respectively. (D) Changes in unnormalized bacterial protein abundance in coculture relative to monoculture shown as a volcano plot as in (C). (E) Changes in quantile-normalized algal protein abundance in coculture relative to monoculture shown as a volcano plot as in (C). Significant differences (triangles) were defined as those where |log2-fold change| > 1, p-adj. < 0.05 from a Welch two sample t-test of the quantile-normalized MASIC values, and the mean MASIC value was greater than the limit of quantitation in both the coculture and the monoculture. Yellow and blue boxes indicate the number of significantly increased and decreased proteins, respectively. (F) Changes in quantile-normalized bacterial protein abundance in coculture relative to monoculture shown as a volcano plot as in (E). (TIF) [file pone.0340253.s002.tif]

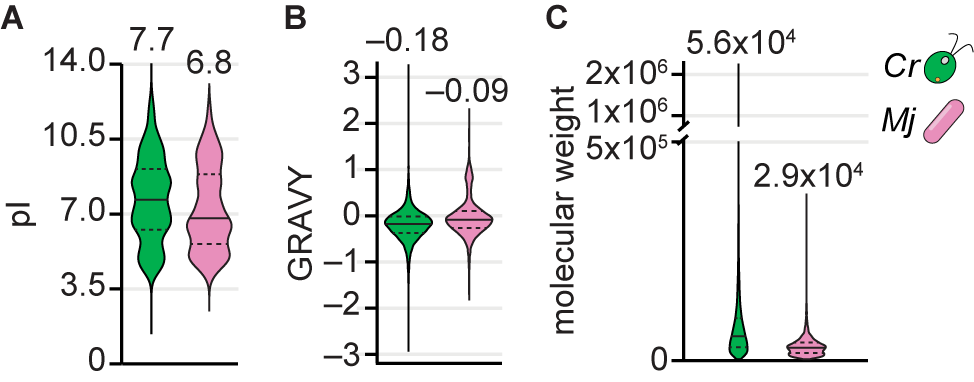

Supplement: S3 Fig — The physicochemical characteristics of C. reinhardtii and M. japonicum proteins were assessed in silico according to Shi et al. 2024 [36]. (A) Distribution of isoelectric points (pI) of proteins encoded by C. reinhardtii (green) and M. japonicum (pink). Median values are represented by the solid horizontal lines and listed above the violins, and quartiles are represented by dashed lines. (B) Distribution of hydropathy (grand average of hydropathy, GRAVY) of proteins encoded by C. reinhardtii (green) and M. japonicum (pink), presented as in (A). (C) Distribution of molecular weights of proteins encoded by C. reinhardtii (green) and M. japonicum (pink), presented as in (A). (TIF) [file pone.0340253.s003.tif]

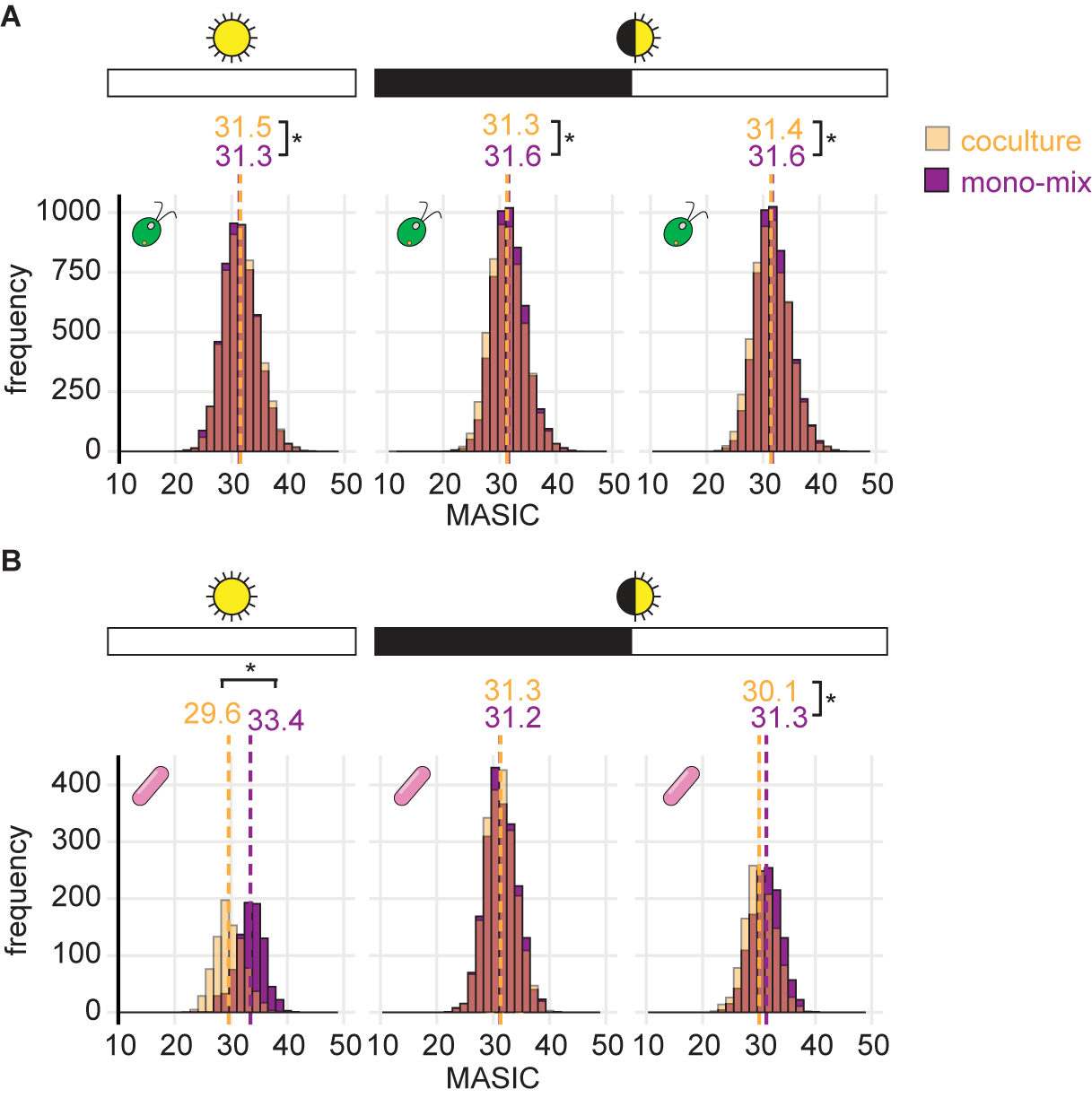

Supplement: S4 Fig — Triplicate cocultures of C. reinhardtii and M. japonicum were grown in parallel with triplicate monocultures of C. reinhardtii and M. japonicum with 150 µg/ml sucrose. Then, the M. japonicum monocultures were added to the C. reinhardtii monocultures to achieve a “mono-mix” control with a similar bacteria-to-algae ratio as the coculture. Cultures were grown in either continuous or diurnal light. Continuous light cultures were collected 36 h after inoculation, and the diurnal light cultures were collected at the end of the night (36 h after inoculation) and the end of the day (48 h after inoculation). (A) Distribution of unnormalized abundances of algal proteins in cocultures (transparent orange) and mono-mix controls (purple) grown in continuous light (sun icon) or diurnal light (eclipsed sun icon) collected at the end of the dark or light phases (black or white bars, respectively). When distributions overlap, the color is muddied. The MASIC values were averaged across the three biological replicates. Median values are shown above the dashed lines. Asterisks next to the medians indicate significant differences between the indicated distributions by a Wilcoxon rank-sum test (p < 0.05). (B) Distribution of unnormalized abundances of bacterial proteins presented as in (A). (TIF) [file pone.0340253.s004.tif]

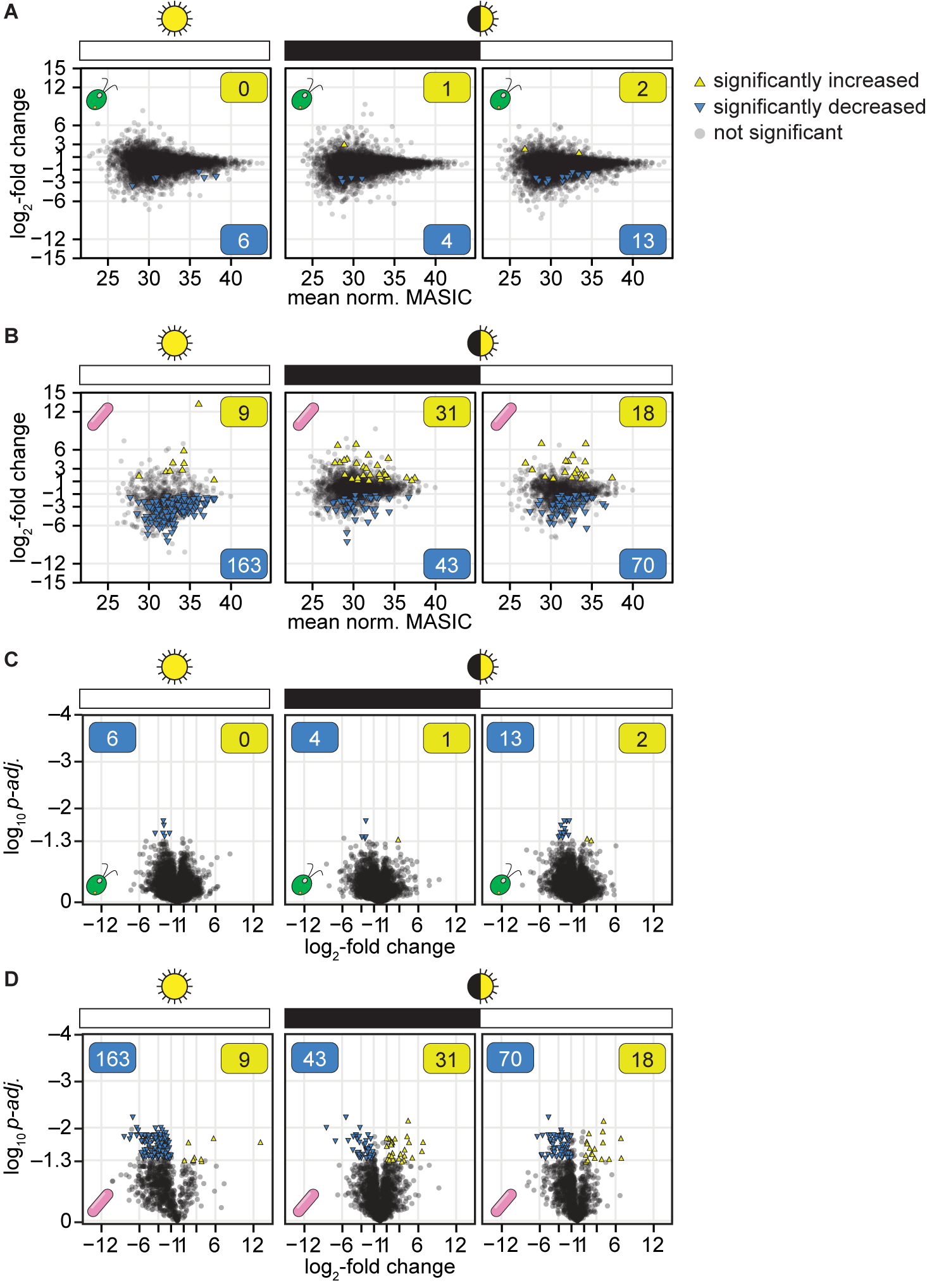

Supplement: S5 Fig — (A) Changes in quantile-normalized algal protein abundances in coculture relative to mono-mix controls when grown in continuous light (sun icon) or diurnal light (eclipsed sun icon) and collected at the end of the dark or light phases (black or white bars, respectively). Significant differences (triangles) were defined as those where |log2-fold change| > 1, p-adj. < 0.05 from a Welch two sample t-test of the quantile-normalized MASIC values, and the mean MASIC value was greater than the limit of quantitation in both the coculture and the monoculture. (B) Changes in quantile-normalized bacterial protein abundances in coculture relative to mono-mix controls presented as in (A). (C) Changes in quantile-normalized algal protein abundances in coculture relative to mono-mix controls when grown in continuous light (sun icon) or diurnal light (eclipsed sun icon) and collected at the end of the dark or light phases (black or white bars, respectively) shown as a volcano plot. Significant differences (triangles) were defined as those where |log2-fold change| > 1, p-adj. < 0.05 from a Welch two sample t-test of the quantile-normalized MASIC values, and the mean MASIC value was greater than the limit of quantitation in both the coculture and the mono-mix controls. (D) Changes in quantile-normalized bacterial protein abundances in coculture relative to mono-mix controls shown as a volcano plot as in (C). (TIF) [file pone.0340253.s005.tif]

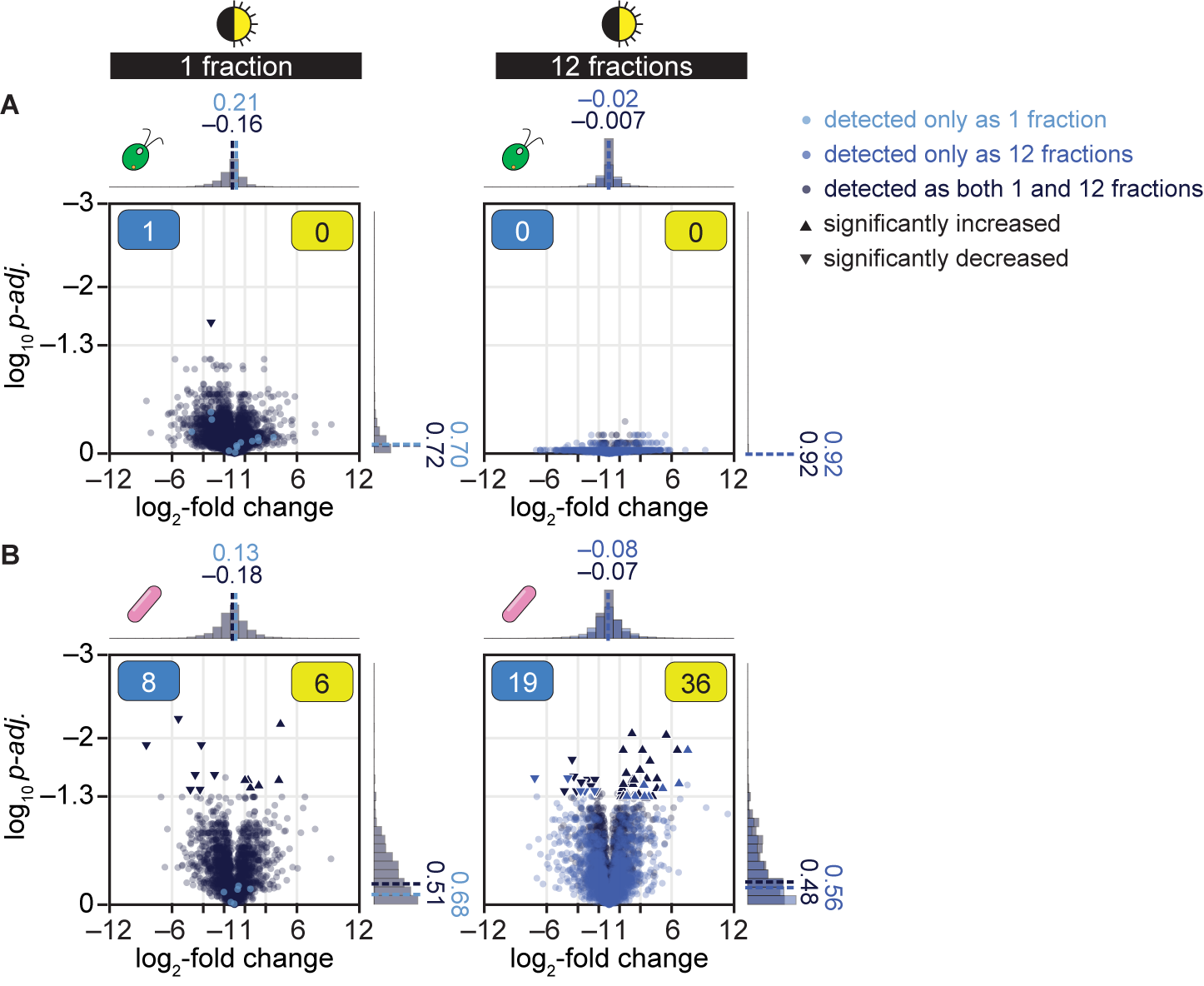

Supplement: S6 Fig — (A) Changes in quantile-normalized C. reinhardtii protein abundance in coculture relative to mono-mix controls at the end of the night when samples were analyzed as 1 (left) or 12 fractions (right). Points are colored based on their detection criteria: lighter blue indicates that the protein only met the criteria when the sample was analyzed as either 1 or 12 fractions, navy blue indicates that the protein met the criteria when the sample was analyzed both as 1 and as 12 fractions. Significant differences (triangles) were defined as those where |log2-fold change| > 1, p-adj. < 0.05 from a Welch two sample t-test of the quantile-normalized MASIC values, and the mean MASIC value was greater than the limit of quantitation in both the coculture and the mono-mix. Yellow and blue boxes indicate the number of significantly increased and decreased proteins, respectively. Histograms show the distribution of log2-fold changes (x axes) and of log10 p-adj. (y axes) for proteins that only met the detection criteria when the sample was analyzed as either 1 or 12 fractions (lighter blue) and for proteins that met the detection criteria when the sample was analyzed both as 1 and as 12 fractions (navy blue); median values are shown above the dashed lines. (B) Changes in quantile-normalized M. japonicum protein abundance in coculture relative to mono-mix controls at the end of the night when samples were analyzed as 1 (left) or 12 fractions (right) presented as in (A). (TIF) [file pone.0340253.s006.tif]
