# Supplementary material for: Mono-mix strategy enables comparative proteomics of a cross-kingdom microbial symbiosis
Source: PLoS One. 2026 Jan 16;21(1):e0340253. doi: 10.1371/journal.pone.0340253 (PMC12810790; doi:10.1371/journal.pone.0340253)
Supplement: Attachment — Submitted filename: Dupuis_PONE_R1_ResponseToReviewers.pdf [file pone.0340253.s015.pdf]

Point-by-Point Response to Referees

Author responses are in blue, manuscript text is in green, line numbers refer to the "clean" manuscript files (R1 = Revision 1, R0 = original submission)

Reviewer #1 comments:

This manuscript is an excellent study that addresses a fundamental and important technical challenge in the proteomics analysis of cross-kingdom microbial symbioses, such as those between algae and bacteria. The problem of abundant host proteins masking the detection of proteins from a less abundant symbiont is a common and significant hurdle for researchers in this field. The authors are to be commended for clearly demonstrating this issue with data, while also presenting practical and effective solutions in the "mono-mix" strategy and sample fractionation. The experimental design is logical, the methodology is appropriate, and the conclusions are well-supported by the data. This work represents an important methodological contribution that will improve the reliability of dual-proteomics analyses of symbiotic systems and is well-suited for publication in PLOS ONE. The manuscript's quality would be further enhanced by addressing the minor revisions detailed below.

We thank the Reviewer for their positive remarks and their suggestions for enhancing the quality of the manuscript.

Minor Comments
To further improve the quality and clarity of the manuscript, I recommend the following minor revisions.

1. Emphasizing the limitations and practical recommendations for the "mono-mix" control
The "mono-mix" strategy is a creative core of this study. However, as the authors frankly acknowledge in the Discussion, a practical challenge is the difficulty in perfectly matching the biomass ratios between the coculture and the mono-mix control. This is the most critical limitation of the method. To ensure readers can implement this strategy without misinterpretation, it would be beneficial to more prominently highlight this limitation and the authors' proposed solutions (e.g., preparing multiple mono-mix ratios, freezing samples for post-hoc mixing) in the Abstract or at the beginning of the Discussion.

We have now demonstrated the limitation of our mono-mix strategy more prominently by 1) alluding to it in the Introduction section (R1 126–128, below), and 2) including a graphic representation of the limitation in a new summary figure that we have added to the Discussion section (R1 Figure 6).

> "We discuss limitations and propose additional improvements to enable robust comparative proteomics of microbial symbioses."

2. Discussion of the protein quantification method
The study uses a "simple rollup" method (summing peptide signal intensities) for protein quantification. While this is a common approach, other quantification algorithms, such as MaxLFQ, are known to be more robust against outlier peptides. A single sentence in the Methods or Discussion briefly explaining the rationale for choosing this method, or acknowledging that other methods could also be applied, would further strengthen the methodological rigor of the paper.

We have now included a sentence explaining why this method was chosen in the Materials and Methods section, R1 L259–267 (new text in **bold**):

> "**To infer protein abundance from peptide abundance, the simple roll-up method was applied, in which** peptide abundances were log_2 transformed, mean central tendency normalized, then grouped by their gene ID and summed per sample, resulting

in protein abundances as log_2-transformed MASIC values. **This common approach was preferred here over other existing approaches for label-free protein quantification (e.g., MaxLFQ [49]), as it is computationally simple, uses all peptide information available, inherently weights signals based on their abundances (i.e., more abundant peptides influence protein abundance determination more than less abundant peptides whose signal may be noisier), and is suitable for LC-MS-level quantitation [50].**"

3. Addition of a summary figure for the overall workflow
This is an excellent methods paper that contrasts a problematic approach with a proposed solution. I strongly recommend adding a schematic summary figure to help readers intuitively grasp the entire workflow. For instance, a figure that visually contrasts the "Conventional Path" (Monoculture vs. Coculture → False Positives) with the "Proposed Path" (Mono-mix + Fractionation vs. Coculture → True Biological Insight) would make the paper's contribution even more clear and impactful.

We thank the reviewer for the excellent suggestion. We now include R1 Figure 6:

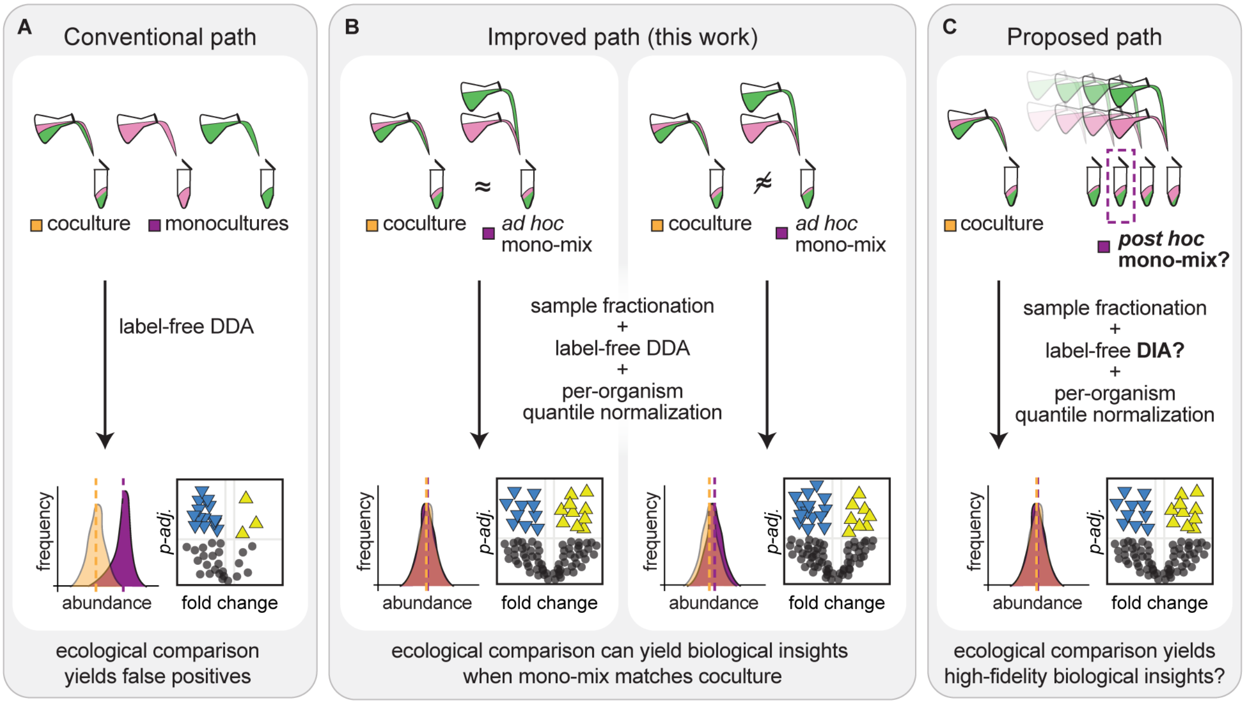

"Fig 6. Graphic representation of challenges in comparative proteomics of microbial symbioses and strategies for overcoming them.

(A) During conventional label-free DDA LC-MS/MS proteomics, increased complexity of coculture samples decreases protein detection in coculture (orange histogram, dotted line represents the median) relative to monoculture (purple histogram, dotted line represents the median). As a result, differential expression analysis of proteins in coculture relative to monoculture results in false-positive proteins with significantly lower abundance in coculture (blue triangles in volcano plot).

(B) In the modified experimental design developed in this work, coculture samples are compared to a "mono-mix" control where upon harvest, monocultures are combined to the predicted relative abundance of the cocultures *ad hoc*. In the improved analytical framework, label-free DDA coverage is increased through peptide sample fractionation, and protein abundance data are quantile normalized per organism. Comparative analysis of the ecological conditions yields biological insights when the relative

abundance of the mono-mix matches that of the coculture (left panel), but false positives arise if it does not (right panel).

(C) For future work, we propose preparation of several mono-mix samples of various target relative abundances upon harvest and *post hoc* selection of the mono-mix whose relative abundance best matches the parallel coculture (dashed purple box). We also propose data-independent acquisition (DIA) LC-MS/MS proteomics to allow unbiased, high coverage of the proteomes of multiple microbes from complex mixed culture samples."

4. A note of caution for the interpretation of Figure 3 results
The PCA in Figure 3D shows that the continuous-light coculture samples (white triangles) are the most distinct from their mono-mix controls (white circles). While this is discussed as a biological response, the data in Figure 2B and 2C show that this same continuous-light condition was the one with the poorest match in biomass ratio between the coculture and the mono-mix. Therefore, the separation seen in the PCA could be influenced not only by biological changes but also by the residual "detection bias." A sentence should be added to the Discussion to acknowledge this as a potential confounding factor when interpreting these specific results.

Indeed, we discussed the PCA separation of the continuous-light coculture samples from their mono-mix controls as methodological rather than biological in R0 L548–558 (R1 L586–596):

> "PCA of *M. japonicum's* proteome showed that the continuous light cocultures (white triangles) were most distinct from their respective mono-mix controls (white circles) (Fig 3D). This was also evident in the *k*-means clustered heatmap, where the majority of bacterial proteins detected appeared less abundant in the cocultures grown in continuous light than in the mono-mix controls grown in continuous light (Fig 3B). As discussed, bacterial protein detection was significantly lower in the continuous light cocultures relative to their mono-mix controls (Figs 2C and 2E). In addition, differential expression analysis suggested that in continuous light, many more proteins were significantly decreased in abundance in coculture than were increased (S5 Fig, S4 Table). Therefore, the patterns observed by clustering analyses likely reflect differences in organism relative abundance and consequent differences in peptide detection in the coculture and mono-mix samples collected from continuous light."

We have now added a warning to this effect in the Discussion section where we instead discuss the impact of *light regime* (rather than coculture vs. monoculture), R1 L773–784 (new text in **bold**):

> "Even though complexity-related differences in peptide detection still influenced the data, the mono-mix experiment revealed that light regime shapes the *M. japonicum* proteome during coculture with *C. reinhardtii*, but not during monoculture (Fig 3). […] Unsurprisingly, we found that the bacterial proteome was distinct in continuous light and diurnal light, likely reflecting more drastic carbon limitation in the latter situation (Fig 3D). Interestingly though, we found that the bacterial proteome is also quite distinct in the day and night phases of the same diurnal period, showing that *M. japonicum's* proteome is shaped by *C. reinhardtii's* diurnal program. **Future work can further disentangle the biological responses from detection-related differences.**"

<u>Reviewer #2 comments:</u>

This is a useful paper that addresses issues that are evident when working with proteomics with two different organisms, and in this case, quite contrasting (prokaryote and eukaryote). The experiments undertaken provide interesting insights into how quantitative information can be more reliable, leading to more confidence in biological interpretations. There are several aspects that need clarifying- such as the issues of imputation and also a more broad assessment of the current status of the field of co-culture proteomics. Specific comments have been added to the manuscript.

We thank Reviewer 2 for the positive remarks. We appreciate their specific comments on statements that could be clarified and suggestions for providing more context to our study.

Specific comments [pasted here from PDF]
Line 121: The introduction is missing a section on what approaches are used to deal with this issue- e.g. cell sorting, normalisation, etc.

We now include such information in the introduction, R1 L99–104:

> "This issue can be avoided by first separating the symbiotic species from one another (e.g., through differential centrifugation) and then analyzing enriched sample fractions by DDA proteomics [37,38]. However, disrupting microbial interactions in this way is less desirable, as separation procedures may change gene expression, and as enriched samples may be biased towards cells that are more loosely associated with their symbionts."

Line 121: Also, info on the selected co-culture is missing- what is known about these strains and specifically their interaction? Biological context is needed here.

Information on Chlamydomonas, Mesorhizobium, and their interaction is included earlier in the introduction section, R0 L66–75 (R1 L66–75):

> "Among the most well studied algae is the unicellular green alga *Chlamydomonas reinhardtii*. This alga is experimentally tractable, can perform both photosynthesis and heterotrophy, and exhibits rhythms in growth and metabolism over diurnal cycles [12,13]. In recent decades, studies have begun to elucidate how *C. reinhardtii* interacts with various bacteria [14–23]. For example, the interaction between the alga and the vitamin B_{12}-producing α-proteobacterium *Mesorhizobium japonicum* has been well documented [15,22,24–26]. This bacterium receives a small but significant amount of photosynthetically fixed carbon in coculture, primarily via algal cell lysis [26]. Yet, it is unknown what specific alga-derived substrates *M. japonicum* and other bacteria may receive from *C. reinhardtii*, and how such partners sense and respond to one another."

Line 128: this isn't clear- what does it mean by signal to noise here? Also, this info on the strain would be better suited to the Introduction.

We have clarified this sentence, R1 L134–136 (new text in **bold**):

> "This strain is amenable to synchronization under diurnal cycles, **yielding populations of cells exhibiting nearly the same physiological state and gene expression, which provide** a system with exceptional signal-to-noise for resolving diurnal patterns [41]."

We have also included this information in the Results section that describes Sample Set 2, providing clearer context for understanding the choice of strains (R1 L481–486):

> "To assess whether these diurnal rhythms influence *M. japonicum* in coculture, we applied the "mono-mix" strategy to cultures grown under continuous light and diurnal light. *C. reinhardtii* strain CC-5390 was used for this experiment, as this strain is amenable to synchronization under diurnal cycles. This yields populations of cells exhibiting nearly the same physiological state and gene expression, which provide exceptional signal-to-noise for resolving diurnal patterns [41]."

Line 130: why modified? Also, any organic carbon in here?

We have revised this sentence of the methods to more clearly describe the medium used for experimentation, which is the same medium used in previous work on this algal-bacterial system, R1 L138–140:

> "Cultures were grown in amended High Salt minimal medium (HSM) [26,42], which contains Kropat's trace element solution [43], plus 2 mM $MgSO_4 \cdot 7H_2O$, 10 µg/l biotin, and 10 µM $CoCl_2$ to support bacterial growth, but lacks a reduced carbon source."

One of the experimental variables was the addition of sucrose (*M. japonicum's* preferred carbon source), which is noted for specific cultures only.

Line 137: scale?

We now include the size of the photobioreactors used, R1 L145–148:

> "For the experiment comparing continuous and diurnal light, algal precultivation occurred in 400 ml flat-panel photobioreactors (Photon System Instruments, Drásov, Czechia) as previously described under the respective light regime to achieve adequate synchrony of the diurnal populations [41]."

Line 166: l should be L (throughout manuscript)

PLOS One submission guidelines state that SI units should be used. The SI brochure, accessible through the link in the PLOS One submission guidelines, explains that:

> "Unit symbols are printed in upright type regardless of the type used in the surrounding text. They are printed in lower-case letters unless they are derived from a proper name, in which case the first letter is a capital letter. An exception, adopted by the 16th CGPM (1979, Resolution 6), is that either capital L or lower-case l is allowed for the litre, in order to avoid possible confusion between the numeral 1 (one) and the lower-case letter l (el)."

Therefore, we have left it as l (el) throughout but are willing to let the copy editor make the change as needed.

Line 214: gap

Fixed from "35cm" to "35 cm."

Line 252: was a median correction applied for quantification?

No additional median correction was applied. Quantile normalization inherently adjusts each sample's distribution, making further median centering unnecessary. We clarified this in the Materials and Methods section (R1 L259–262 & L276–281):

> "To infer protein abundance from peptide abundance, the simple roll-up method was applied, in which peptide abundances were $\log_2$ transformed, mean central tendency normalized, then grouped by their gene ID and summed per sample, resulting in protein abundances as $\log_2$-transformed MASIC values. […] Log_2-transformed protein MASIC values were quantile normalized using the normalize.quantiles function from the R package *preprocessCore* (v1.66.0) [51,52]. Quantile normalization was performed independently for each organism: the distributions of *C. reinhardtii* protein abundances across the relevant samples were quantile normalized together, and the distributions of *M. japonicum* protein abundances across the relevant samples were quantile normalized together."

Line 269: What imputation algorithm was used? Were missing values considered?

Missing values were handled by exclusion rather than imputation. We re-organized the relevant portion of the Materials and Methods section and added text to this effect to make this clearer, R1 L268–275 & L287–293 (new text in **bold**, deleted text ~~struck through~~):

> "Only proteins with at least two unique peptides for quantitation that were detected in at least two of the three biological replicates in any condition were analyzed. In the unfractionated pilot experiment (Sample Set 1), this included 9871 proteins (6113 *C. reinhardtii* proteins, 3758 *M. japonicum* proteins). In the unfractionated mono-mix experiment (Sample Set 2), this included 9784 proteins (7039 *C. reinhardtii* proteins, 2745 *M. japonicum* proteins). In the mono-mix experiment comparing protein detection before and after peptide sample fractionation (Sample Set 2, fractionated), this included 15,464 proteins (10,858 *C. reinhardtii* proteins, 4606 *M. japonicum* proteins). **Missing values were handled by exclusion rather than imputation.**
> […]
> The average protein abundances were calculated after excluding ~~non-detected~~ **missing** values and the $\log_2$-fold change in protein abundance was calculated as (mean abundance in coculture) – (mean abundance in monoculture). To calculate the Z-score abundance for each protein in a given experiment, ~~non-detected~~ **missing** values were excluded, then the mean and standard deviation of quantile-normalized MASIC values for each protein across the experimental conditions was calculated, and the Z-score was calculated as (mean norm. MASIC – (mean of all mean norm. MASIC))/(sd of all mean norm. MASIC)."

Line 383: Doesn't seem very surprising- can quantitative data be added to give more of an insight? Also, what about other factors such as database sizes- do these have any impact?

Quantitative data from our reanalysis of proteomics data from two published studies are represented in S1 Figure and the associated statistical tests cited in the Results section (R0 L368–379, R1 L395–407). These quantitative data include changes in the numbers of unique proteins detected across the various sample types and the significant differences in the distributions of protein abundances in coculture vs. monoculture that we observed upon reanalysis of the two published studies. These were the metrics by which we reported sample-complexity-related differences in protein detection in our own datasets.

The Reviewer also points out database size as a potentially important consideration. The impact of database size for microbial coculture proteomics was explored previously by Shi *et al*. 2024 J. Proteome Res. (Ref. 36). This study found that although searching to a larger database decreased the number of proteins identified due to increased false discovery rate, use of a large, concatenated database still allowed for identification of 98% of proteins and is thus suitable.

We note that for our experiments included in the present manuscript, data from all samples was searched against the same concatenated database. Since the database size and other qualities remained constant, they could not have contributed to the decrease in bacterial protein detection observed in coculture samples relative to monoculture samples in the present study.

We have now:

1) Clarified that data from all samples were searched against the same database and included information about database sizes of the genome annotation files used for *C. reinhardtii* and *M. japonicum* in the Materials and Methods, R1 L238–244 (new text in **bold**):

   "**All data** were searched against a single concatenated FASTA file containing amino acid sequences from both the *C. reinhardtii* reference genome assembly and annotations v6.1 (https://phytozome-next.jgi.doe.gov/info/CreinhardtiiCC_4532_v6_1) **(32,670 protein entries, 17,691 protein-coding genes)** [44], the *M. japonicum* MAFF303099 genome assembly and annotations (https://www.ncbi.nlm.nih.gov/datasets/genome/GCF_000009625.1/) **(7,105 protein entries, 7,162 protein-coding genes)**, and commonly observed contaminant proteins **(16 protein entries).**"

2) Included information about database sizes of the genome annotation files used for in the previously published studies that we explored in the figure legend of S1 Fig (new text in **bold**):

   "Number of unique *Arthrobacter* proteins with at least two peptides detected in at least one biological replicate of monocultures (purple) and cocultures with *C. reinhardtii* (orange) 19 h and 168 h after inoculation by LC-MS/MS proteomics reported in Windler *et al.* 2022. **Spectra were mapped to the *C. reinhardtii* v5.6 genome annotation (17,741 protein-coding genes) and *Arthrobacter* strain P2b genome annotation (4,475 protein-coding genes).** […] Number of unique *Dehalococcoides ethenogenes* proteins detected in at least two biological replicates of monocultures (purple) and cocultures with *Desulfovibrio vulgaris* (orange) by LC-MS/MS proteomics reported in Men *et al.* 2012. **Spectra were mapped to a concatenated database of amino acid sequences from *D. ethenogenes* strain 195 (~1,500 protein-coding genes) and *D. vulgaris* strain Hildenborough (~3200 protein-coding genes).**"

3) Drawn attention to database size in the Results section where the Reviewer made the suggestion, R1 L399–404 (new text in **bold**):

   "We wondered if decreased detection of bacterial proteins in coculture samples was unique to those containing eukaryotic partners with larger cells, or rather generalizable to any sample with another microbe. Therefore, we also reanalyzed label-free DDA LC-MS/MS proteomics data from syntrophic bacterial cultures of *Dehalococcoides*

ethenogenes strain 195 and *Desulfovibrio vulgaris* Hildenborough [54]. In this case, the two organisms are closer in **both cell size and the number of protein-coding genes**."

Line 398: Were missing values checked here?

We have now specified how missing values were handled in the Materials and Methods section describing statistical testing of significant differences in protein abundance, R1 L318–326 (new text in **bold**):

> "Significant differences in the abundance of a given protein in coculture relative to monoculture were assessed using the t.test function in the R package *stats* (v4.4.0). Only proteins that were detected in at least two of the three biological replicates in both the monoculture control condition and the respective coculture condition were analyzed. In the unfractionated pilot experiment (Sample Set 1), this included 5368 *C. reinhardtii* proteins and 1737 *M. japonicum* proteins. In the unfractionated mono-mix experiment (Sample Set 2), it included 6246 *C. reinhardtii* proteins and 2128 *M. japonicum* proteins. In the fractionated mono-mix experiment (Sample Set 2, fractionated), it included 9934 *C. reinhardtii* proteins and 4179 *M. japonicum* proteins). **Missing values were handled by exclusion rather than imputation.**"

Line 406: where is this method from and why was this one chosen?

We now include the appropriate references for quantile normalization and the R package used to implement them in the Materials and Methods section (Ref. 51–52), and we explain choice of this approach in the Results section, R1 L432–436:

> "We applied quantile normalization, a complete normalization strategy that equalizes sample distributions by ranking the values in each sample and substituting the values by the rank-wise means [51]. This strategy has been shown to optimally reduce technical variability in shotgun LC-MS/MS datasets [56]."

Line 438: grammar

We have revised to the following, R1 L471–473 (new text in **bold**, deleted text ~~struck through~~):

> "We ~~conceived of~~ **developed** a modified experimental design that we term the "mono-mix" strategy, in which DDA peptide detection in control samples may more closely emulate detection in coculture samples."

Line 684: does this have implications for what was reported biologically?

We re-analyzed two previously published proteomics studies: Windler *et al.* and Men *et al.*. Windler *et al*. did not use their proteomics data to investigate significant changes to the bacterial proteome in coculture relative to monoculture. In private communications with the authors, they stated that analysis of the bacterial proteomics data was not included in their publication due to sparse detection of the bacterial proteins in coculture, especially when compared to monoculture. Their publication only commented on what the most abundant bacterial proteins detected were. Thus, complexity-related differences in detection would likely not have implications for what was reported in this case.

Men *et al*. did use their proteomics data to investigate the impact of symbiosis on *D. ethenogenes'* proteome. For example, they say in the discussion "The down-regulation of these genes [in coculture relative to monoculture] suggests that the syntrophic growth of DE195 and DVH might have a different hydrogen transfer system than the DE195 isolate." It is possible that the "downregulated" proteins mentioned here could be a detection-related false positive of differential expression analysis, one that yields a misleading hypothesis for future work.

Line 687: Can this be clarified with more details? Line 690: which part of Fig 1?

We have clarified with more details and specified the relevant figure panels, R1 L748–751 (new text in bold):

> "**Quantile** normalization **per organism** led to greater overlap in the distributions of protein abundances **in coculture samples and monoculture samples (Figs 1C–1D). However**, differential expression analysis using quantile-normalized values still suggested that most bacterial proteins were significantly decreased in abundance in coculture relative to monoculture (Fig 1**E**)."

Line 696: state the ratio used here

We include additional text here to make clearer that the bacteria-to-algae ratio used for the mono-mix controls depends on which coculture sample they would be compared to, R1 L755–759 (new text in **bold**):

> "we developed a modified experimental design that we term the "mono-mix" strategy, where *C. reinhardtii* monocultures and *M. japonicum* monocultures are combined into a single mono-mix control that emulates the bacteria-to-algae ratio of the coculture sample **to which they will be compared (Figure 6B)**. Using this strategy, detection of bacterial proteins was more comparable in cocultures and monoculture **controls** (Fig 2)."

Line 703: explain what "reductive division" is.

We now define reductive division, R1 L764–766 (new text in **bold**):

> "…as *M. japonicum* undergoes reductive division when in coculture with *C. reinhardtii* [26]. **The bacterium continues to divide despite reduced biomass synthesis, resulting in smaller cells.**"

Line 719: re-write, what is shaped? Proteome/metabolism ??

We now specify, R1 L781–783 (new text in **bold**):

> "Interestingly though, we found that the bacterial proteome is also quite distinct in the day and night phases of the same diurnal period, showing that *M. japonicum***'s proteome** is shaped by *C. reinhardtii's* diurnal program."

Line 739: Could more stringent cut offs reduce false positives?

False positives in differential expression analysis in this context arise from reduced protein detection in coculture relative to monoculture, rather than changes in reproducibility that might affect significance testing. We demonstrate by including volcano plots (R1 S2C–F Figs, R1

S5C–D Figs) showing the distribution of *p. adj* values to complement the abundance vs. fold-change plots we show.

Line 755: what are the implications when more complex communities are being investigated- like 3 or 4 strains compared to metaproteomics?

We state in the Discussion section, R0 L737–740 (R1 L801–804):

> "Thus, we predict that in addition to monocultures and cocultures, any two treatments that exhibit substantial differences in the relative abundance of interacting organisms would suffer from altered detection by DDA that could result in false positives during differential expression analysis."
